# Prognostic Value and Related Molecular Mechanisms of miR‐5003‐3p in Hepatocellular Carcinoma

**DOI:** 10.1155/cjgh/1318790

**Published:** 2026-04-05

**Authors:** Hao Yan, Wei Shan, Qian Xia, Wanting Fang

**Affiliations:** ^1^ Department of Interventional Vascular Surgery, General Hospital of Northern Theater Command, Shenyang, 110001, China, syjqzyy.com; ^2^ Department of Pathology, Beifang Hospital of China Medical University, General Hospital of Northern Theater Command, Shenyang, 110001, China, syjqzyy.com

**Keywords:** hepatocellular carcinoma, MAL2, miR-5003-3p, prognosis, progression

## Abstract

**Background:**

Hepatocellular carcinoma (HCC) is a malignant tumor worldwide with a high mortality rate and recurrence rate. Numerous miRNAs are being applied to the healing and prognosis of HCC. A prior investigation predicted that miR‐5003‐3p was linked to HCC, but the relevant molecular mechanisms were not clear.

**Aim:**

To discover the prognostic value and molecular mechanisms concerned of miR‐5003‐3p in HCC.

**Methods:**

A total of 125 tumor specimens from HCC patients were obtained in this study, along with corresponding adjacent noncancerous tissue samples collected as a control. The levels of miR‐500‐3p and MAL2 in tumor tissues and cells were detected by RT‐qPCR. The prognostic value of miR‐500‐3p was evaluated using Kaplan–Meier curve and COX regression model. The effects of miR‐500‐3p on cellular malignant phenotypes were assessed via CCK‐8 and Transwell assays. The target sites of miR‐500‐3p were identified using bioinformatics analysis. The dual‐luciferase reporter assay validated the target relationship between them.

**Results:**

miR‐5003‐3p levels were remarkably elevated in HCC tissues, and late TNM stage (I + II) was dramatically higher versus early TNM stage (III + IV). Lymph node metastasis, TNM stage, and differentiated degree were linked notably to miR‐5003‐3p expression. Upregulated miR‐5003‐3p was an independent risk factor for HCC. In vitro, downregulated miR‐5003‐3p could induce apoptosis and restrain proliferation, migration, and invasion, which could be rescued by depressed MAL2.

**Conclusion:**

miR‐5003‐3p may be an independent prognostic factor for HCC. Mechanistically, miR‐5003‐3p negatively regulates MAL2 to promote cellular processes. These findings highlight the potential of miR‐5003‐3p as a novel prognostic biomarker and a promising therapeutic target for HCC.

## 1. Introduction

Primary liver cancer is a malignant tumor prevalent in the gastrointestinal system, with the third highest mortality rate among malignant tumors globally [1]. Hepatocellular carcinoma (HCC) occupies 90% of primary liver cancers, making it the most predominant pathologic type [2, 3]. Major risk factors for HCC involve chronic hepatitis C virus (HCV) or hepatitis B virus (HBV), heavy alcohol consumption, and diabetes [4–6]. Currently, liver transplantation (LT) and liver resection (LR) are the mainstay of curing HCC. As it allows complete removal of the lesion, LT is the optimum therapy and offers the best prognosis. The postoperative 5 year survival rate for LT is 75%, with a 20% recurrence rate [7, 8]. However, due to the shortage of liver donors, LR is more common [9, 10]. LR could dramatically prolong the survival of patients with early‐stage HCC, providing a 5 year postoperative survival rate of 50%, but with a recurrence rate of up to 70% [11–13].

A growing volume of research is applying miRNAs to the diagnosis and treatment for HCC. It has been proposed that miRNAs may serve as diagnostic factors and prognostic markers in multiple tumors, which are engaged in regulating the levels of oncogenes and tumor suppressor genes [14]. Several dysregulated miRNAs were discovered in HCC, which were engaged in various processes [15, 16]. For instance, high levels of miR‐4465 and miR‐134 promoted apoptosis and depressed proliferation in HCC cells [17, 18]. miR‐424‐5p and miR‐143‐3p could modulate migration and invasion [19, 20]. Moreover, miR‐101‐3p and miR‐1246 were implicated in epithelial mesenchymal transition in HCC [15, 21]. The levels of miR‐5003‐3p were lower in healthy paracancerous tissue and higher in cervical cancer tissue [22]. And it has also been detected to be upregulated in breast cancer cells [23]. Moreover, miR‐5003‐3p could promote the malignant phenotype of cervical cancer [22]. Inhibition of miR‐5003‐3p suppressed epithelial‐mesenchymal transition, migration, and invasion in breast cancer cells [23]. This indicated that highly expressed miR‐5003‐3p may promote cancer progression. Notably, bioinformatics results revealed that miR‐5003‐3p was overexpressed in HCC [24]. Yet no specific clinical trials underpin this, and the molecular mechanisms are not clear. In this article, miR‐5003‐3p was uncovered for significance in the HCC clinic and relevant molecular mechanisms.

## 2. Materials and Methods

### 2.1. Study Subjects

Approval for this research was obtained from the Ethics Committee of the General Hospital of the Northern Theater Command. The trial covered 125 patients with HCC diagnosed between 2020 and 2021. All patients underwent surgical resection without prior antitumor therapy. We collected paired HCC tissues as well as normal paracancerous tissues from our patients. Resected tissues were verified by at least two pathologists and stored at −80°C for further study. All patients had acquired informed consent and were monitored for survival for 5 years after surgery.

### 2.2. Cell Lines

Immortalized human hepatic cell line (THLE‐2) and HCC cell lines (Hep3B, Huh‐7, SNU‐398, Li‐7) were sourced from Shanghai Cell Bank (Shanghai, China). DMEM (Gibco, USA) medium containing 10% FBS was employed to culture cells with 5% CO_2_ at 37°C.

### 2.3. Cell Transfection

The miR‐5003‐3p mimic and miR‐5003‐3p inhibitor (RiboBio, China) were designed to overexpress or suppress miR‐5003‐3p level in HCC cells. Mimic NC and inhibitor NC (RiboBio, China) were negative controls. MAL2 siRNA (Si‐MAL2) (RiboBio, China) was employed to repress MAL2 expression, while si‐NC (RiboBio, China) was a negative control. Hep3B and Huh‐7 cells (2 × 10^5^ cells/well) were seeded into 6‐well plates and cultured for 24 h. Subsequently, 100 nM miR‐5003‐3p mimic, miR‐5003‐3p inhibitor, or 100 nM si‐MAL2 was transfected into cells using Lipofectamine 2000 (ThermoFisher, USA). Cells were harvested 48 h post‐transfection.

### 2.4. Real‐Time Quantitative PCR (RT‐qPCR)

Total RNA was extracted from tissues or HCC cells via Trizol reagent (Takara, Japan) and was reverse transcribed to cDNA with the PrimeScript RT Master Mix kit (Takara, Japan). The RT‐qPCR assay was run on a 7500 Rapid Real‐Time PCR System using BeyoFast SYBR Green qPCR Mix (Beyotime, China), employing the manufacturer‐recommended 20 μL reaction volume. U6 and GAPDH were utilized as internal references for miR‐5003‐3p and MAL2, respectively. The levels of miR‐5003‐3p and MAL2 in HCC tissues or cells were calculated using the 2^−ΔΔCt^ method. The primer sequences used were as follows (5′ to 3′): miR‐5003‐3p forward primer: ACACTCCAGC TGG​GTA​CTT​TTC​TAG​GTT​G; reverse primer: TGGTG TCGTGGAGTCG. MAL2 forward primer: GAC​ATC​CTG​CGG​ACC​TAC​TC; reverse primer: AGA​CAA​GAC​CCC​CGA​ACA​G.

### 2.5. Dual‐Luciferase Reporter Assay

By using TargetScanHuman8.0 (https://www.targetscan.org/vert_80/), we obtained the binding sequences for miR‐5003‐3p and MAL2. Subsequently, nucleic acid sequences covering the binding site and corresponding mutant sequences were amplified and inserted into the pmirGLO vector (Promega, USA) to construct wild‐type (pmirGLO‐MAL2‐WT) or mutant (pmirGLO‐MAL2‐MUT) expression plasmids. The above plasmids were co‐transfected with miR‐5003‐3p mimic or miR‐5003‐3p inhibitor into Hep3B and Huh‐7 cells. 48 h later, luciferase activity was assessed by the Dual Luciferase Reporter Kit (Promega, USA), using the system recommended by the manufacturer.

### 2.6. Flow Cytometry Analysis

We employed FITC Annexin V Apoptosis Detection Kit II (BD Bioscience, USA) to measure apoptosis of HCC cells. Firstly, cells were digested using trypsin (ThermoFisher, USA) and washed with PBS. Subsequently, they were fixed with 70% ethanol and incubated at 4°C overnight. After washing with PBS, cells were stained by 5 μL propidium iodide and 5 μL Annexin V‐FITC solution for 30 min under a light‐avoidance environment. Afterwards, apoptosis was checked by FACS Caliber flow cytometer (BD Bioscience).

### 2.7. CCK‐8 Assay

Transfected Hep3B and Huh‐7 cells (5 × 10^3^ cells/well) were seeded into 96‐well plates and cultured at 37°C. After 24, 48, and 72 h, 10 µL of CCK8 solution (Solarbio, China) was added to each well and incubated for 2 h. The absorbance at 450 nm was measured for each well.

### 2.8. Transwell Assay

The Corning Tranwell 24‐well plates were utilized for migration assays. Added 300 μL of serum‐free DMEM containing cells (1 × 10^5^ cells/well) to the upper chamber. Added 500 μL of DMEM containing 20% FBS but no cells to the lower chamber. Incubated the plates at 37°C for 24 h. Subsequently, the upper chamber was removed, gently washed with PBS, and placed in 4% paraformaldehyde (Sigma, Germany) to fix for 30 min. It was then stained with 0.1% crystal violet (Sigma, Germany) for 20 min. After staining, excess dye was washed off with PBS, and the upper layer of cells was gently scraped off with a cotton swab. The upper chamber was inverted to air‐dry naturally, and cell counts were performed under a microscope.

The procedure for the invasion assay was identical to that of the migration assay. The only difference was that prior to the experiment, we added 50 μL of BD Biocoat Matrigel Matrix (BD Bioscience, USA) to the upper chamber and allowed it to stand at 37°C for 2 h.

### 2.9. Statistical Analysis

Data were handled with IBM SPSS Statistics 27 and GraphPad Prism 9, which were denoted as mean ± SD. The Kaplan–Meier curve with the log‐rank test was employed to examine survival rate. The chi‐square test was applied to evaluate the connection of gene expression with clinicopathological features. Differences within two cohorts were tested by *t*‐test. Multisets were checked through one‐way ANOVA. Pearson’s correlation analysis visualized the correlation for miR‐5003‐3p with MAL2. *p* < 0.05 was regarded as a significant difference.

## 3. Results

### 3.1. Expression and Prognostic Value of miR‐5003‐3p in HCC Patients

We gathered HCC tissues along with paracarcinoma tissues from 125 patients of different TNM periods. The experimental procedure was shown in Supporting Figure 1. The research process included: (1) collection of clinical samples and detection of miR‐5003‐3p expression; (2) prognostic analysis and correlation with clinical characteristics; (3) cellular functional experiments; (4) target gene verification; and (5) rescue experiments.

RT‐qPCR results indicated that the levels of miR‐5003‐3p were increased by nearly 0.5‐fold in HCC tissues compared to adjacent noncancerous tissues (*p* < 0.001; Figure 1(a)). Based on the average expression levels of miR‐5003‐3p in tumor tissues, patients were divided into a high‐expression group (≥ 1.40) and a low‐expression group (< 1.40). Comparison of the clinicopathological features between the two groups revealed that miR‐5003‐3p expression was notably correlated with lymph node metastasis (*p* < 0.05), TNM stage (*p* < 0.01), and differentiated degree (*p* < 0.05) (Table 1). According to the TNM stage, the expression of miR‐5003‐3p in tumor tissues (relative to adjacent noncancerous tissues) was subjected to stratified analysis. We found that the miR‐5003‐3p expression increased by approximately 0.3‐fold in advanced‐stage (TNM stage III + IV) patients compared to early‐stage (TNM stage I + II) patients (*p* < 0.001; Figure 1(b)).

FIGURE 1Prognostic value and expression of miR‐5003‐3p in HCC. (a) miR‐5003‐3p levels were dramatically elevated in cancer than adjacent. ^∗∗∗^ *p* < 0.001. (b) miR‐5003‐3p expression was remarkably enhanced in late TNM (III + IV) versus early TNM (I + II). ^∗∗∗^ *p* < 0.001. (c) Survival rate in high miR‐5003‐3p group was inferior to low miR‐5003‐3p group.

**Figure:**
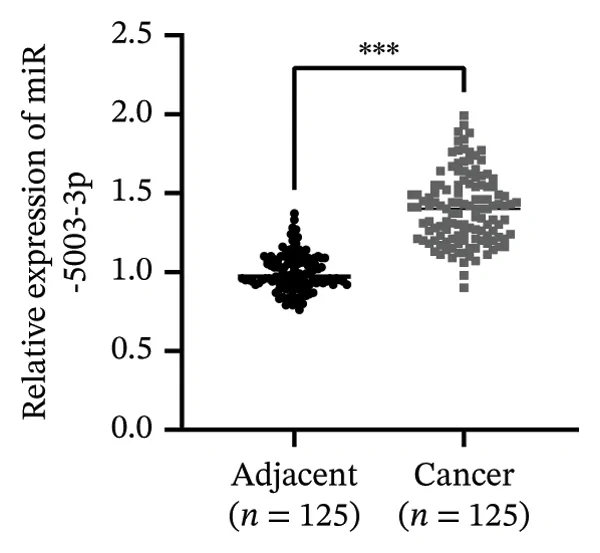
(a)

**Figure:**
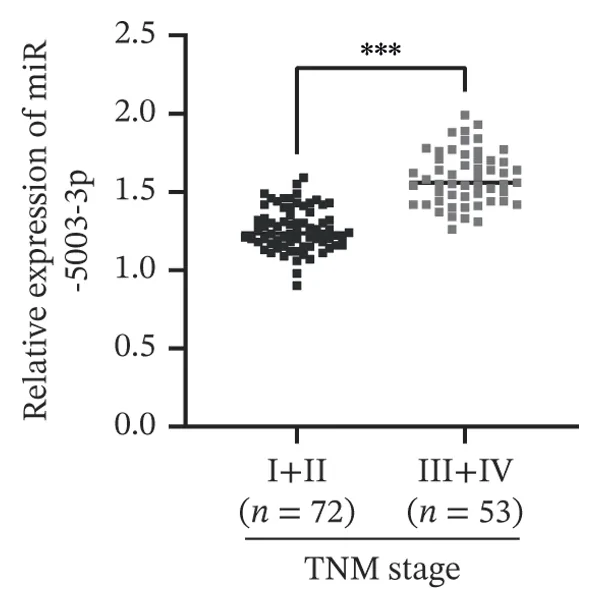
(b)

**Figure:**
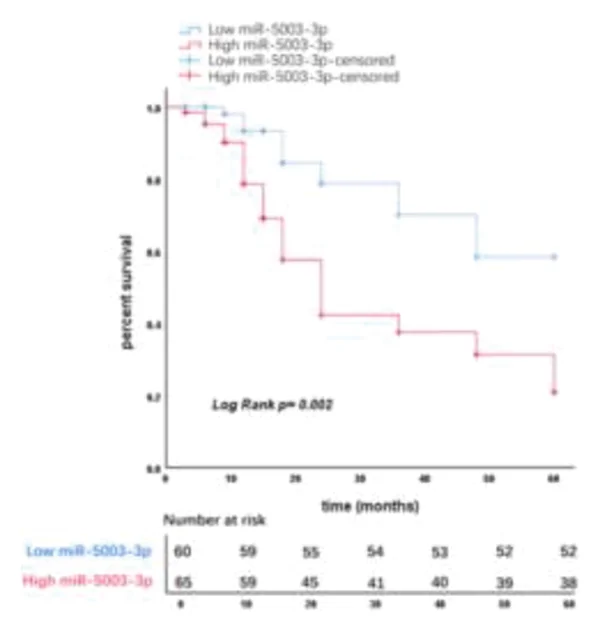
(c)

**TABLE 1:** Relationship between miR‐500‐3p expression and clinicopathological features for HCC.

Characteristics	miR‐5003‐3p expression		*p* value
	Low (*n* = 60)	High (*n* = 65)	
Age (years)	57.73 ± 7.85	58.43 ± 8.53	0.636
Gender			0.568
male	32	38	
female	28	27	
HBV infection (case)			0.554
positive	40	40	
negative	20	25	
Liver cirrhosis (case)			0.312
presence	35	32	
absence	25	33	
Diameter of tumor (cm)			0.073
< 5	40	33	
≥ 5	20	32	
Lymph node metastasis			0.011^∗^
yes	18	34	
no	42	26	
TNM stage (case)			0.007^∗∗^
I + II	42	30	
III + IV	18	35	
Differentiated degree (case)			0.043^∗^
well/moderate	42	34	
poor	18	31	

Abbreviation: HBV, hepatitis B virus.

^∗^ *p* < 0.05.

^∗∗^ *p* < 0.01.

The Kaplan–Meier curve uncovered that patients with higher miR‐5003‐3p levels had shorter survival (Log Rank *p* = 0.002; Figure 1(c)). The outcome of Cox analysis denoted miR‐5003‐3p (*p* < 0.05), lymph node metastasis (*p* < 0.05), and TNM stage (*p* < 0.01) as high‐risk factors contributing to HCC patients’ progression (Table 2).

**TABLE 2:** Multivariate Cox analysis of prognostic parameters in patients with HCC.

Variable	HR	95% CI for HR		*p* value
		Lower	Upper	
miR‐5003‐3p	2.777	1.126	6.851	0.027^∗^
Age	1.082	0.498	2.353	0.842
Gender	1.492	0.715	3.117	0.287
HBV infection	1.120	0.509	2.464	0.778
Liver cirrhosis	1.462	0.679	3.150	0.332
Diameter of tumor	2.161	0.959	4.872	0.063
Lymph node metastasis	2.561	1.172	5.597	0.018^∗^
TNM stage	2.145	1.006	4.574	0.048^∗^
Degree of differentiation	2.091	1.928	4.709	0.075

Abbreviation: HBV, hepatitis B virus.

^∗^ *p* < 0.05.

### 3.2. Effect of miR‐5003‐3p on Cellular Processes in HCC Cells

In vitro, we assessed the expression of miR‐5003‐3p in HCC cell lines. It was observed that miR‐5003‐3p levels were notably higher in Hep3B (*p* < 0.001), Huh‐7 (*p* < 0.001), SNU‐398 (*p* < 0.01), and Li‐7 (*p* < 0.01) than in THLE‐2 (Figure 2(a)). Thus, we opted for Hep3B and Huh‐7 in our subsequent experimental investigations. By transfecting miR‐5003‐3p mimic, miR‐5003‐3p inhibitor, or their negative controls (mimic NC or inhibitor NC) into Hep3B and Huh‐7 cells, our findings discovered that miR‐5003‐3p mimic promoted miR‐5003‐3p levels (*p* < 0.001) whereas miR‐5003‐3p inhibitor repressed miR‐5003‐3p expression (*p* < 0.01, *p* < 0.001; Figure 2(b)). Moreover, miR‐5003‐3p mimic enhanced proliferation (*p* < 0.01) and repressed apoptosis (*p* < 0.01), whilst miR‐5003‐3p inhibitor repressed proliferation (*p* < 0.01) and fostered apoptosis (*p* < 0.01; Figures 2(c), 2(d), and 2(e)). Migration and invasion were facilitated by upregulated miR‐5003‐3p (*p* < 0.001) and suppressed by downregulated miR‐5003‐3p (*p* < 0.01, *p* < 0.001; Figures 2(f), and 2(g)). In summary, overexpression of miR‐5003‐3p promotes the malignant phenotype of HCC cells.

FIGURE 2Impact of miR‐5003‐3p expression on HCC cells. (a) miR‐5003‐3p expression was markedly above in Hep 3B, Huh‐7, SNU‐398 and Li‐7 than THLE‐2. ^∗∗^ *p* < 0.01, ^∗∗∗^ *p* < 0.001. (b) miR‐5003‐3p mimic dramatically facilitated miR‐5003‐3p levels. miR‐5003‐3p inhibitor dramatically inhibited miR‐5003‐3p levels. ^∗∗^ *p* < 0.01, ^∗∗∗^ *p* < 0.001. (d‐d) Upregulated miR‐5003‐3p promoted proliferation markedly. Downregulated miR‐5003‐3p repressed proliferation considerably. ^∗∗^ *p* < 0.01. (e) miR‐5003‐3p mimic strikingly inhibited apoptosis while miR‐5003‐3p inhibitor stridently induced apoptosis. ^∗∗^ *p* < 0.01. (f‐g) Upregulated miR‐5003‐3p enhanced migration and invasion. Downregulated miR‐5003‐3p repressed migration and invasion. ^∗∗^ *p* < 0.01, ^∗∗∗^ *p* < 0.001.

**Figure:**
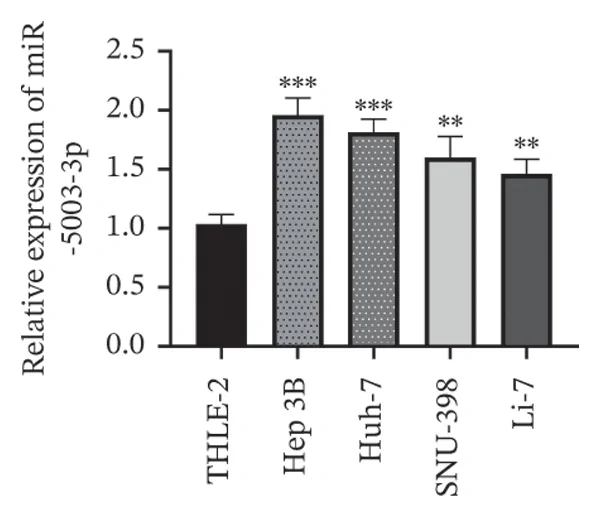
(a)

**Figure:**
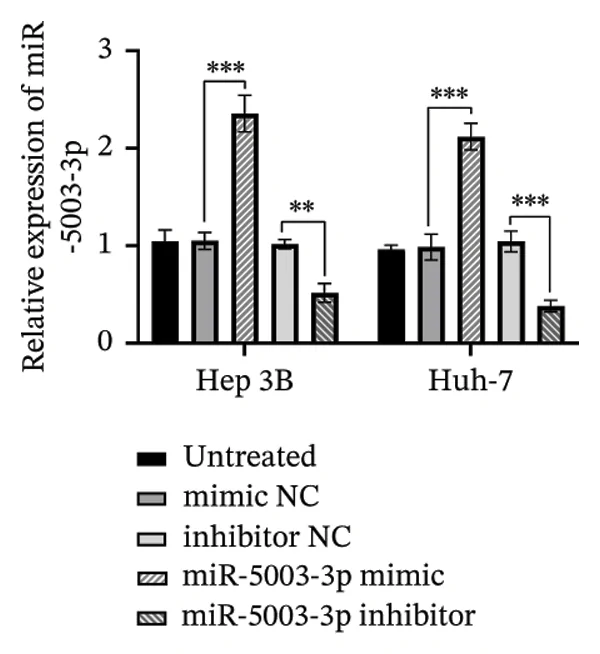
(b)

**Figure:**
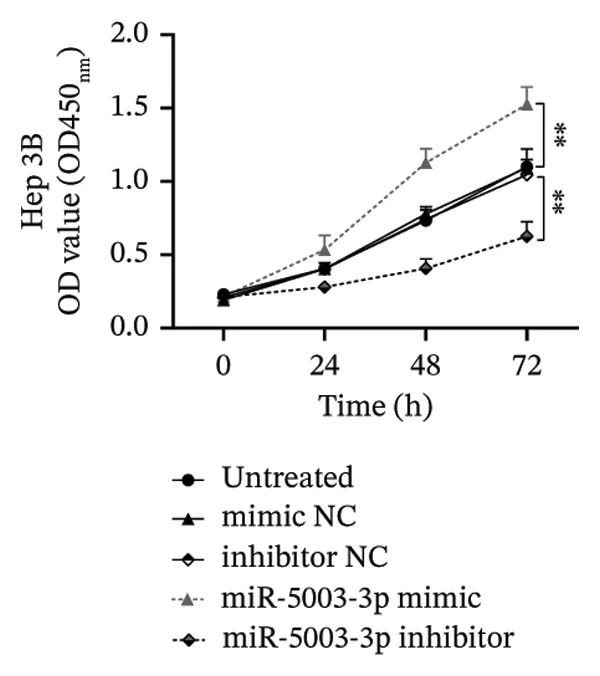
(c)

**Figure:**
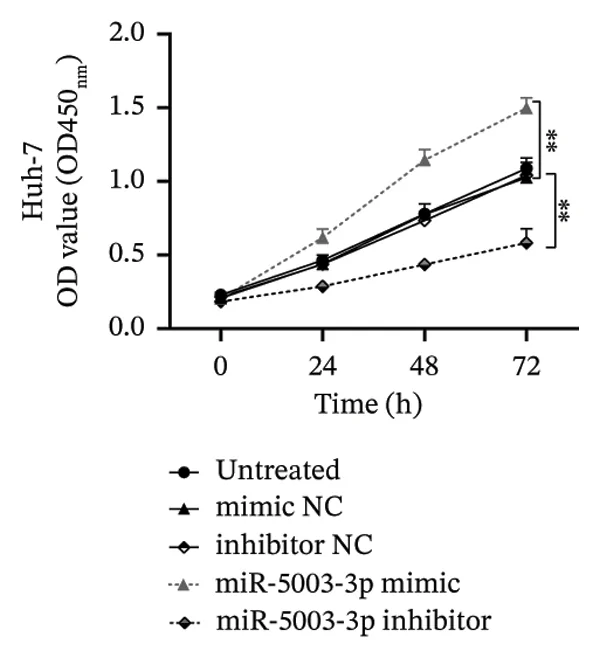
(d)

**Figure:**
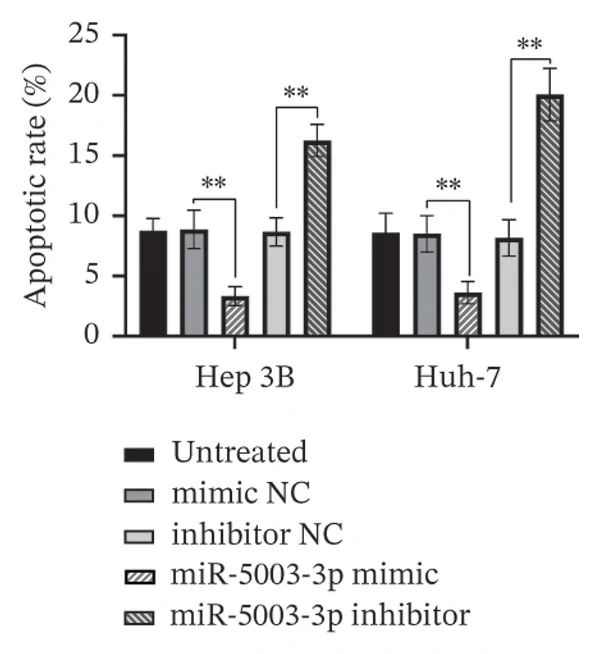
(e)

**Figure:**
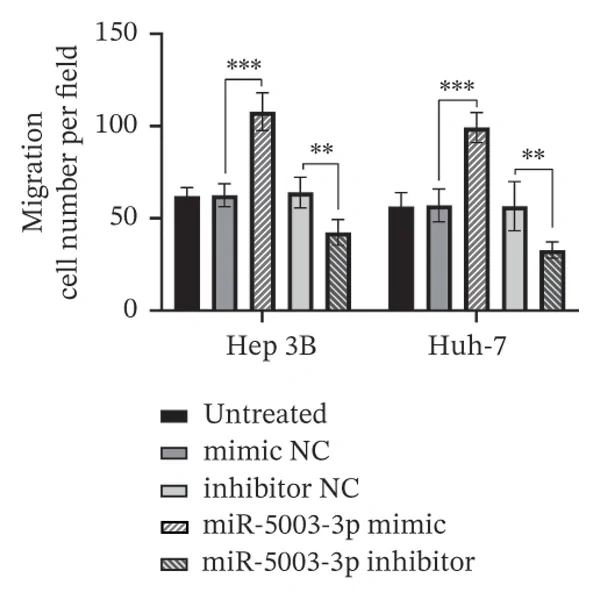
(f)

**Figure:**
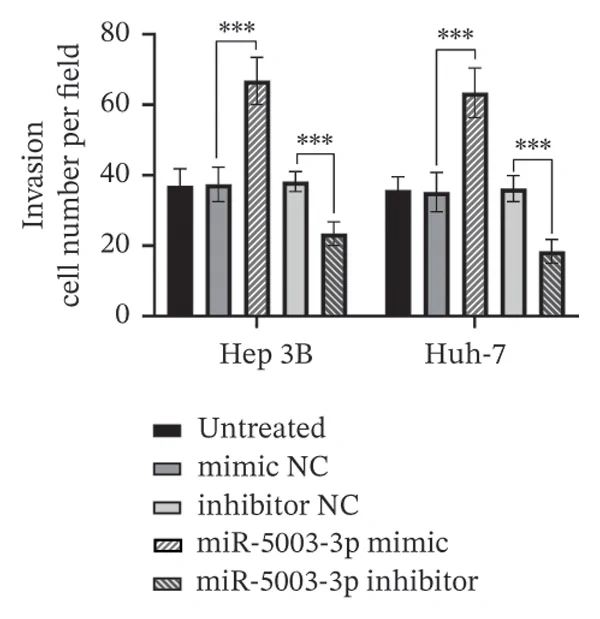
(g)

### 3.3. MAL2 is a Target Gene of miR‐5003‐3p

Bioinformatics analysis revealed that miR‐5003‐3p has a binding site with MAL2 (Figure 3(a)). In the MAL2‐WT group, miR‐5003‐3p mimic suppressed the luciferase activity (*p* < 0.01), whereas miR‐5003‐3p inhibitor exerted the opposite effect (*p* < 0.01, *p* < 0.001). No significant changes were observed in the MAL2‐MUT group (Figures 3(b), and 3(c)). In HCC cells, miR‐5003‐3p mimic suppressed MAL2 expression while miR‐5003‐3p inhibitor enhanced MAL2 expression (*p* < 0.01, *p* < 0.001; Figure 3(d)). In patients, the levels of MAL2 in HCC tissue were reduced by approximately 0.3‐fold compared to adjacent noncancerous tissue (*p* < 0.001; Figure 3(e)). And it was decreased by 0.3‐fold in advanced‐stage (TNM stage III + IV) patients compared to early‐stage (TNM stage I + II) patients (*p* < 0.001; Figure 3(f)). Besides, Pearson correlation analysis exhibited a strong negative correlation of miR‐5003‐3p and MAL2 (*p* < 0.001; Figure 3(g)). In brief, MAL2 is a target gene of miR‐5003‐3p and is negatively regulated by miR‐5003‐3p.

FIGURE 3Association of miR‐5003‐3p with MAL2. (a) Binding site of miR‐5003‐3p to MAL2. (b‐c) miR‐5003‐3p mimic strikingly suppressed MAL2 levels. miR‐5003‐3p inhibitor strikingly promoted MAL2 levels. ^∗∗^ *p* < 0.01, ^∗∗∗^ *p* < 0.001. (d) MAL2 expression in miR‐5003‐3p mimic transfected cells was decreased than control. MAL2 expression in miR‐5003‐3p inhibitor transfected cells was elevated than control. ^∗∗^ *p* < 0.01, ^∗∗∗^ *p* < 0.001. (e) MAL2 levels were substantially lower in cancer versus adjacent. ^∗∗∗^ *p* < 0.001. (f) MAL2 expression was markedly decreased in late TNM (III + IV) versus early TNM (I + II). ^∗∗∗^ *p* < 0.001. (g) miR‐5003‐3p was dramatically negatively correlated with MAL2.

**Figure:**
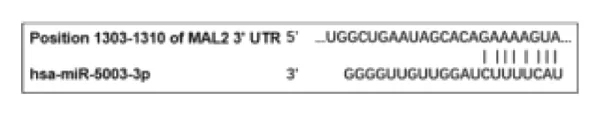
(a)

**Figure:**
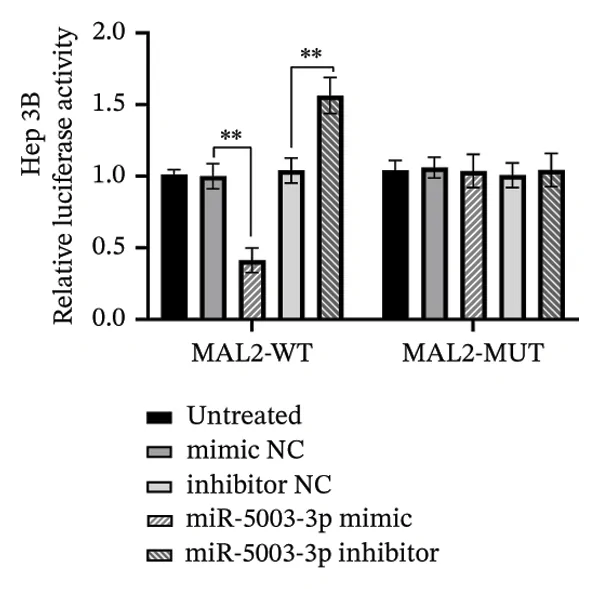
(b)

**Figure:**
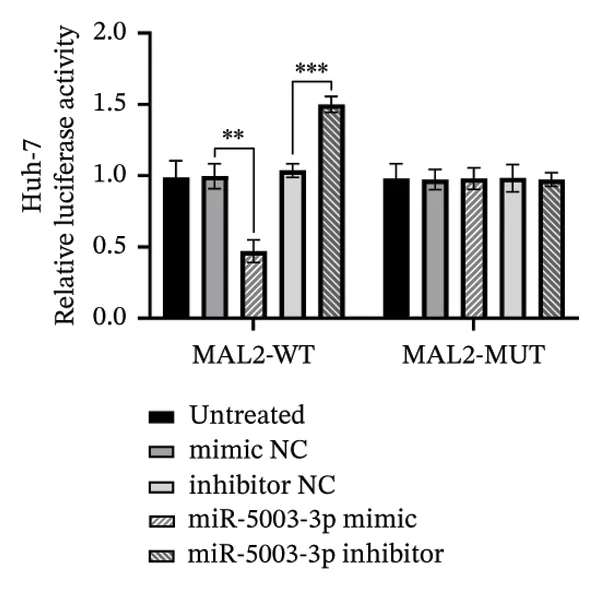
(c)

**Figure:**
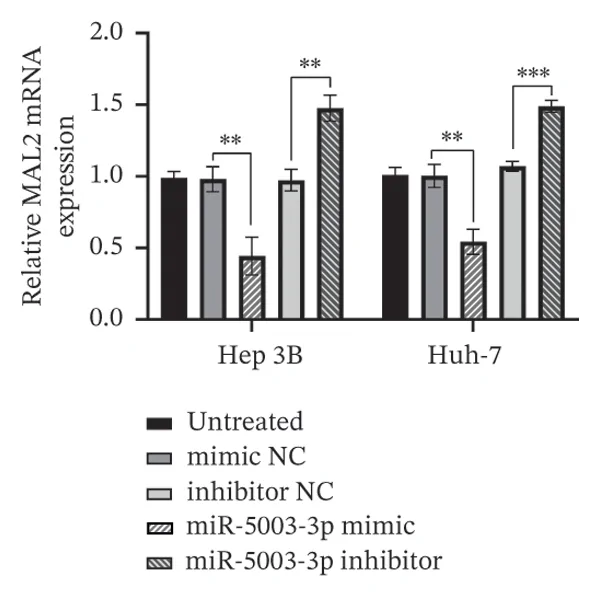
(d)

**Figure:**
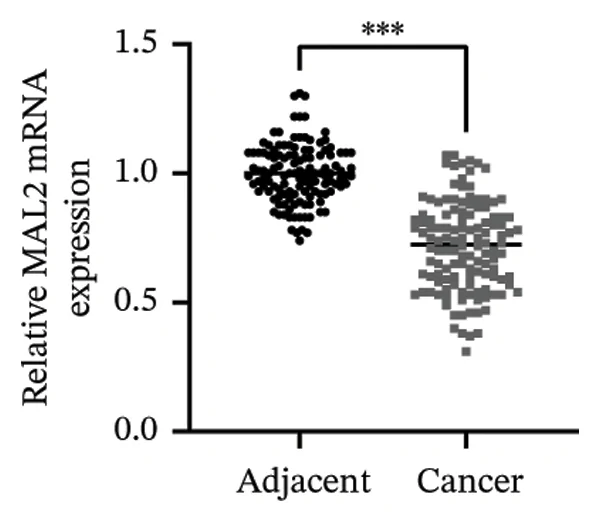
(e)

**Figure:**
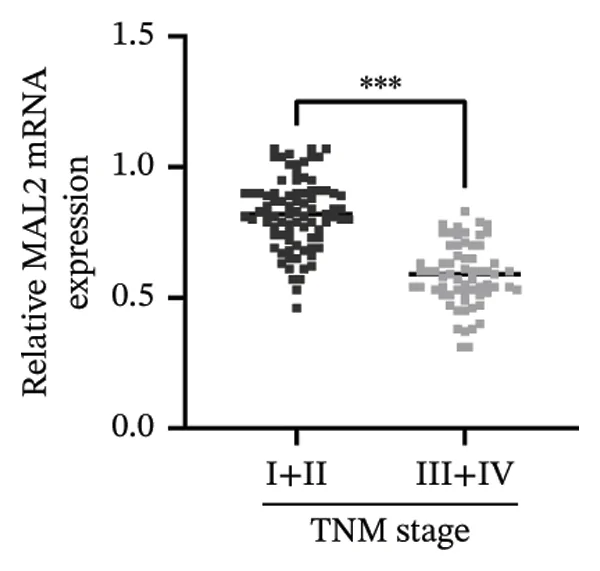
(f)

**Figure:**
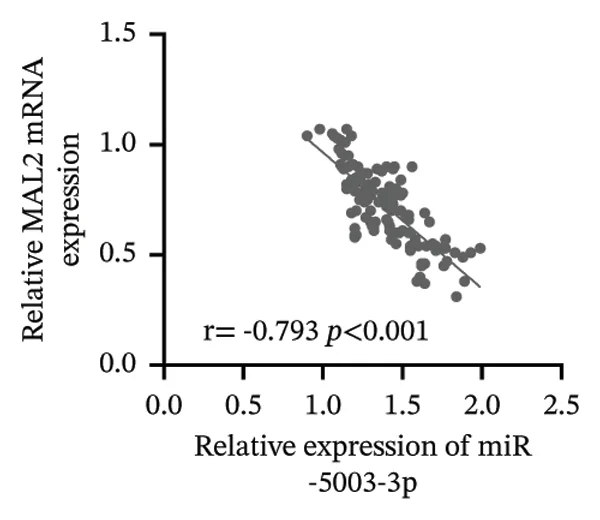
(g)

### 3.4. MiR‐5003‐3p Impacts Cellular Processes by Negatively Regulating MAL2

After validating the miR‐5003‐3p and MAL2 targeting relationship, we conducted a series of functional rescue experiments. It was noticed that downregulated miR‐5003‐3p remarkably enhanced MAL2 expression (*p* < 0.01), and the phenomenon could be rescued by inhibiting MAL2 (*p* < 0.01, *p* < 0.001; Figure 4(a)). Furthermore, the miR‐5003‐3p inhibitor dramatically inhibited proliferation (*p* < 0.001) and promoted apoptosis (*p* < 0.001, *p* < 0.01), which could be reversed by inhibition of MAL2 expression (*p* < 0.01; Figures 4(b), 4(c), and 4(d)). Similarly, the miR‐5003‐3p inhibitor notably suppressed cell migration and invasion (*p* < 0.001), which was rescued by downregulated MAL2 (*p* < 0.001; Figures 4(e) and 4(f)). Consequently, repressed miR‐5003‐3p suppresses the malignant phenotype of HCC cells by negatively regulating MAL2.

FIGURE 4Effect of miR‐5003‐3p and MAL2 combined on HCC. (a) Elevated MAL2 expression due to miR‐5003‐3p inhibitor was reversed by downregulation of MAL2. ^∗∗^ *p* < 0.01, ^∗∗∗^ *p* < 0.001. (b‐c) Downregulated miR‐5003‐3p restrained proliferation, which could be rescued by inhibiting MAL2. ^∗∗^ *p* < 0.01, ^∗∗∗^ *p* < 0.001. (d) Low level of miR‐5003‐3p facilitated apoptosis, which could be reversed by downregulation of MAL2. ^∗∗^ *p* < 0.01, ^∗∗∗^ *p* < 0.001. (e‐f) miR‐5003‐3p inhibitor suppressed migration and invasion, which could be rescued by repressing MAL2. ^∗∗∗^ *p* < 0.001.

**Figure:**
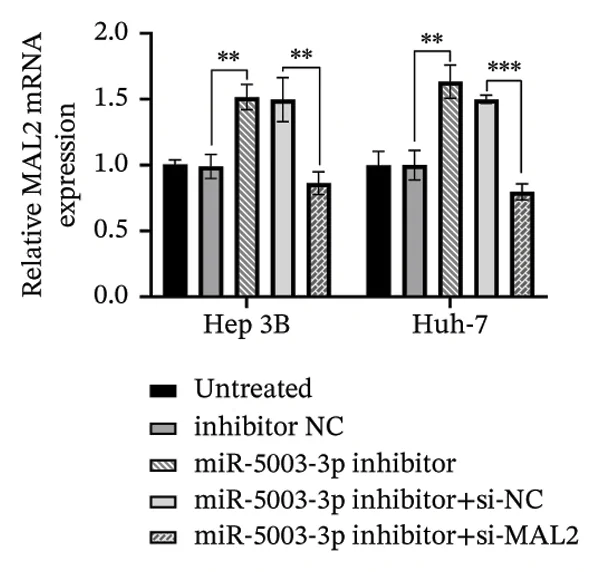
(a)

**Figure:**
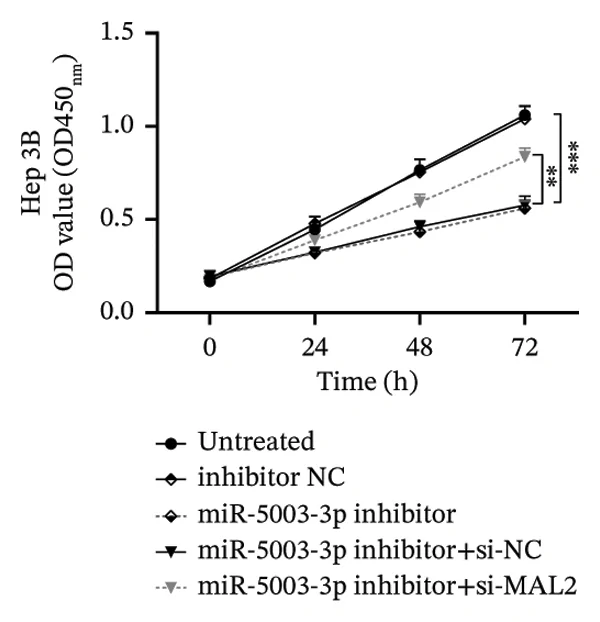
(b)

**Figure:**
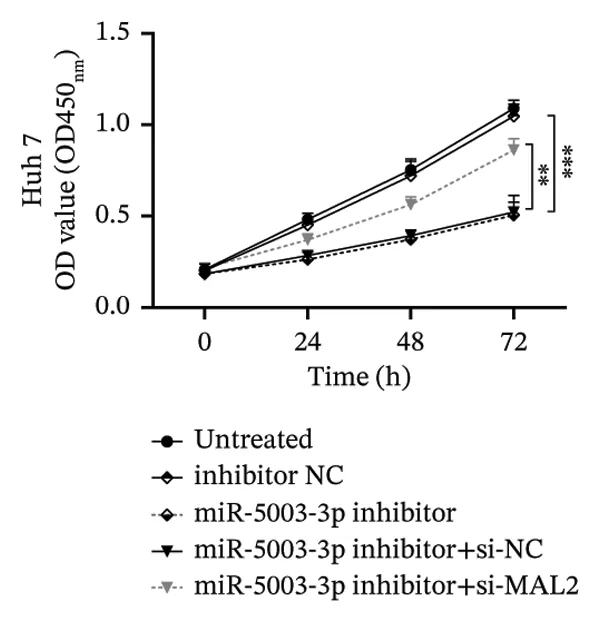
(c)

**Figure:**
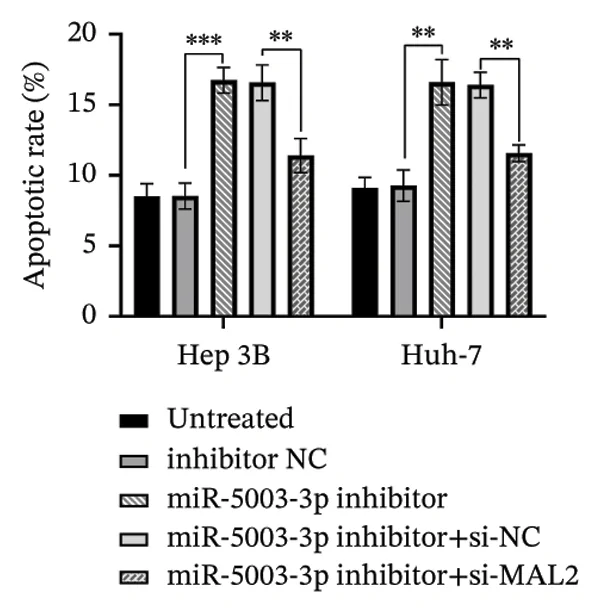
(d)

**Figure:**
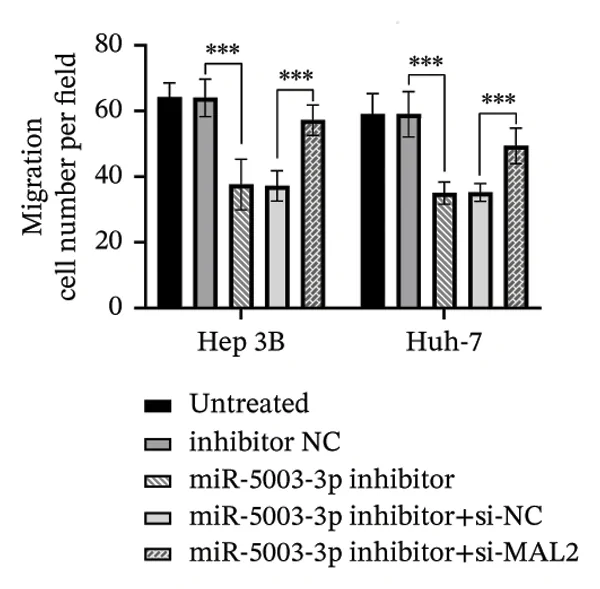
(e)

**Figure:**
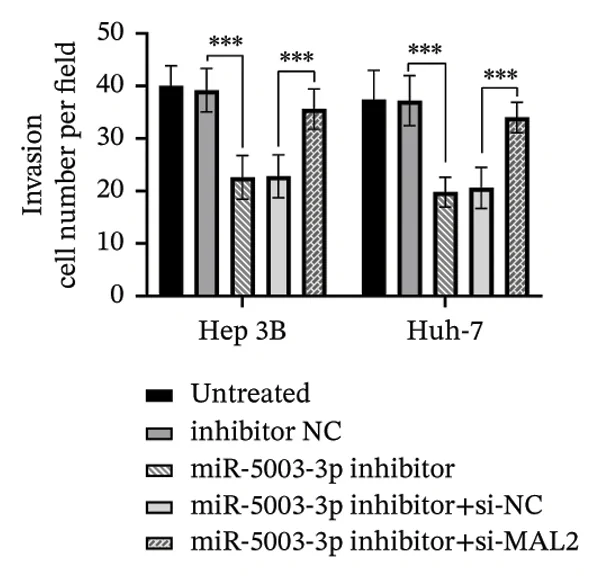
(f)

## 4. Discussion

Liver cancer is a widespread malignant neoplasm worldwide, with a high mortality rate [25]. HCC is the primary type of liver cancer, whose main risk factor is chronic viral hepatitis caused by HBV and HCV infections [5, 26]. Currently, therapies for HCC range from LT, LR, embolization, and radiation to systemic anticancer therapy, etc. [27]. Optimal therapy varies for patients at different periods. Owing to clinical diagnostic challenges in the early stage, the majority of patients were denied the diagnosis until the mid‐ to late‐stage and miss the optimal time for treatment. Moreover, median overall survival in HCC patients ranged from 5 years in the early stage to 3 months in the advanced stage, which portends a poor prognosis [27]. A prior study discovered that miR‐5003‐3p was differentially distributed in HCC versus normal tissues by bioinformatics analysis [24]. And upregulated miR‐5003‐3p has been identified in both cervical and breast cancers [22, 23]. Therefore, we hypothesized that miR‐5003‐3p may involve in HCC progression. In this trial, HCC tissues from clinical patients were assembled and analyzed. MiR‐5003‐3p levels were identified to be substantially elevated in tumor tissues and were increased in advanced TNM stage (III + IV) compared to early stage (I + II). Meanwhile, higher levels of miR‐5003‐3p were accompanied by shorter survival. Cox regression analysis uncovered that miR‐5003‐3p was an independent prognostic factor for HCC. Hence, we considered that miR‐5003‐3p may serve as a prognostic biomarker for HCC.

Rising studies have applied miRNAs to the diagnosis and therapy of various cancers including HCC [28, 29]. Numerous miRNAs have been proven to be dysregulated in HCC, which impacts tumor regression [15, 16, 20]. It has been discovered that overexpressed miR‐5003‐3p facilitated cellular progression of cervical cancer cells, whereas downregulated miR‐5003‐3p prevented migration and invasion of metastatic breast cancer cells [22, 23]. Similarly, our findings uncovered that upregulated miR‐5003‐3p could enhance proliferation, migration, and invasion and dampen apoptosis, whereas inhibition of miR‐5003‐3p could restrain proliferation, migration, and invasion and foster apoptosis. The above outcomes indicated that miR‐5003‐3p promoted the malignant phenotype of HCC cells, which laterally corroborated our hypothesis.

To probe molecular mechanisms involved in miR‐5003‐3p, MAL2 was filtered. MAL2 is in the MAL family, which acts as an integral part of membrane transport [30]. Prior work revealed that MAL2 remarkably declined in HCC, cholangiocarcinoma, and renal cell carcinoma by bioinformatics analysis [31]. Additionally, MAL2 overexpression in colorectal cancer strikingly repressed cell proliferation and invasion [32]. Such evidence suggests that MAL2 might be a tumor suppressor, which is consistent with our results. We noticed that MAL2 expression in HCC tissues was markedly below and decreased in advanced TNM stages versus early stages. Suppression of MAL2 levels could rescue the induction by downregulated miR‐5003‐3p in repression of proliferation, migration, and invasion along with induction of apoptosis. The action of MAL2 in tumor suppression may be linked to the induction of actin remodeling of filamentous pseudopods to reduce migratory, invasive and proliferative effects [31, 33]. Accordingly, we speculated that downregulated miR‐5003‐3p may induce the formation of cellular filopodia by negatively regulating MAL2, thereby inhibiting proliferation, migration, and invasion, which in turn mitigates HCC progression.

It has been established that MAL2 was engaged in modulating several signaling pathways. As an example, overexpression of MAL2 could activate the MAPK/mTOR signaling pathway in non‐small‐cell lung cancer, contributing to active ribosome biogenesis and enhancing cell proliferation [34]. In intrahepatic cholangiocarcinoma, MAL2 could stabilize EGFR membrane localization and stimulate PI3K/AKT/SREBP‐1 pathway to enhance lipid metabolism [35]. Moreover, MAL2 could exert influence on the biological processes of breast cancer through the β‐catenin/c‐Myc axis [36]. It is notable that these pathways still fulfill vital roles in HCC [37]. Hence, to elucidate the downstream signaling mechanisms of MAL2 and further refine the regulatory network of the miR‐5003‐3p/MAL2 axis, we plan to employ techniques such as Western blot analysis to detect the expression of key proteins (e.g., *p*‐ERK and *p*‐AKT) in these pathways following MAL2 regulation. Intriguingly, overexpressed MAL2 was uncovered in numerous tumors, such as cervical and breast cancers [36, 38]. The reasons behind the differing expression of MAL2 in various tumors are not clear.

The limitations of this study were as follows: Firstly, as a single‐center study, it had limitations in sample representativeness and the generalizability of conclusions. In the future, we will expand both the sources and size of our sample to ensure the scientific validity and persuasiveness of our findings. Secondly, the mechanism research was derived solely from cell experiments and lacked in vivo validation. We will initiate in vivo experiments as soon as possible. Thirdly, the prognostic value of miR‐5003‐3p should be further evaluated in large external datasets (such as TCGA or GEO). In short, our study discovered that miR‐5003‐3p may be an independent prognostic factor for HCC, offering clinical samples to support a previous study. Mechanistically, miR‐5003‐3p facilitates HCC cellular processes by negatively regulating MAL2. It provides a possible therapeutic target for HCC therapy going forward.

## Funding

No funding was received to assist with the preparation of this work.

## Conflicts of Interest

The authors declare no conflicts of interest.

## Supporting Information

Supporting Figure 1. The flowchart of the experiment in the article.

## Supporting information


**Supporting Information** Additional supporting information can be found online in the Supporting Information section.

## Data Availability

The datasets used and/or analyzed during the current study are available from the corresponding author on reasonable request.
