# Supplementary material for: Prognostic Value and Related Molecular Mechanisms of miR‐5003‐3p in Hepatocellular Carcinoma
Source: Can J Gastroenterol Hepatol. 2026 Apr 5;2026:1318790. doi: 10.1155/cjgh/1318790 (PMC13580813; doi:10.1155/cjgh/1318790)
Supplement: Supplementary file 1 [file CJGH-2026-1318790-s001.pdf]

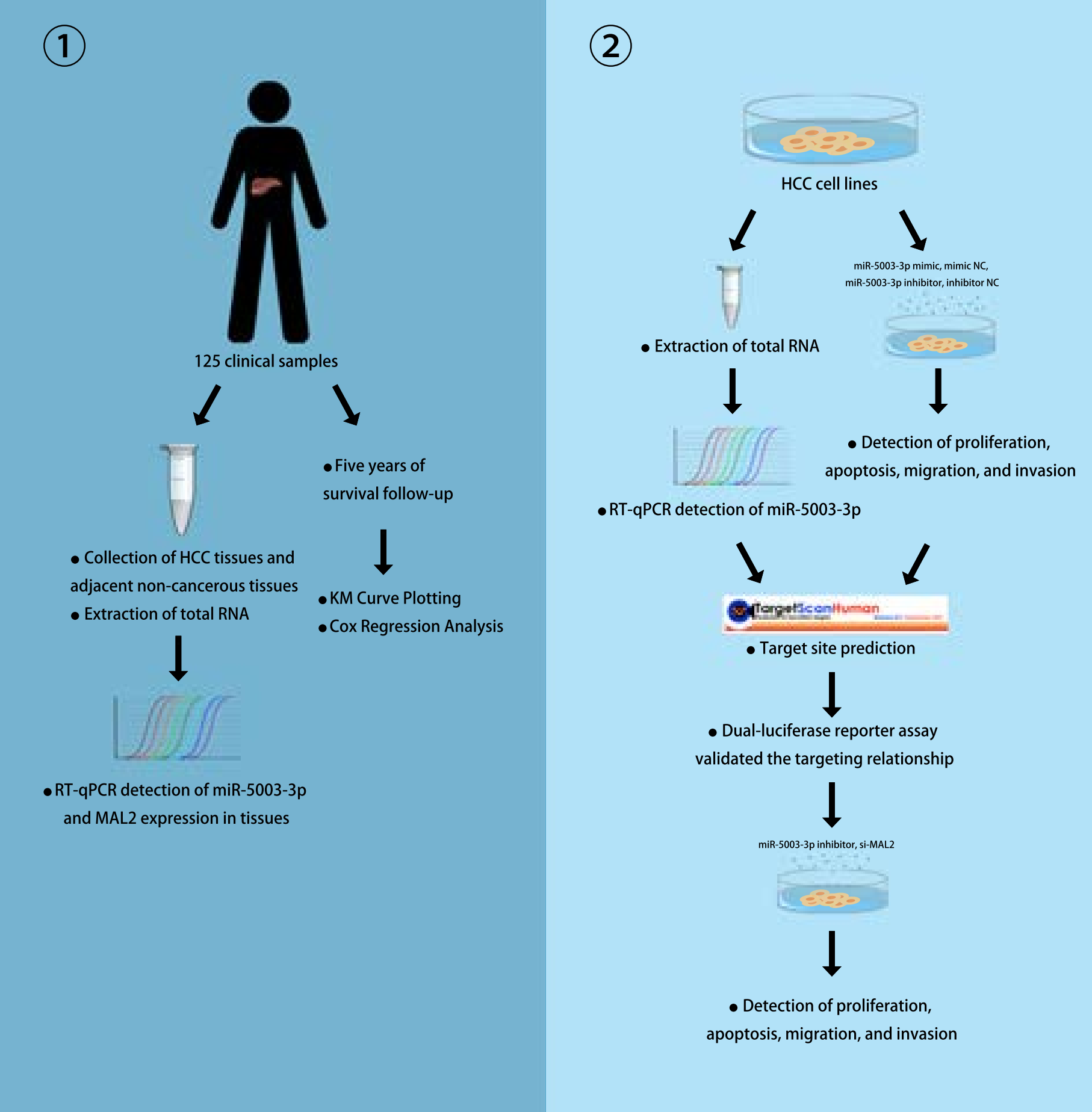
①
125 clinical samples
● Collection of HCC tissues and
adjacent non-cancerous tissues
● Extraction of total RNA
● RT-qPCR detection of miR-5003-3p
and MAL2 expression in tissues
● Five years of
survival follow-up
● KM Curve Plotting
● Cox Regression Analysis
②
HCC cell lines
● Extraction of total RNA
● RT-qPCR detection of miR-5003-3p
miR-5003-3p mimic, mimic NC,
miR-5003-3p inhibitor, inhibitor NC
● Detection of proliferation,
apoptosis, migration, and invasion
TargetScanHuman
● Target site prediction
● Dual-luciferase reporter assay
validated the targeting relationship
miR-5003-3p inhibitor, si-MAL2
● Detection of proliferation,
apoptosis, migration, and invasion
